# Reinforcement Learning-Based Joint Beamwidth and Beam Alignment Interval Optimization in V2I Communications

**DOI:** 10.3390/s24030837

**Published:** 2024-01-27

**Authors:** Jihun Lee, Hun Kim, Jaewoo So

**Affiliations:** Department of Electronic Engineering, Sogang University, Seoul 04107, Republic of Korea

**Keywords:** vehicle communications, antenna beamwidth, beam alignment overhead, beam alignment interval, reinforcement learning

## Abstract

The directional antenna combined with beamforming is one of the attractive solutions to accommodate high data rate applications in 5G vehicle communications. However, the directional nature of beamforming requires beam alignment between the transmitter and the receiver, which incurs significant signaling overhead. Hence, we need to find the optimal parameters for directional beamforming, i.e., the antenna beamwidth and beam alignment interval, that maximize the throughput, taking the beam alignment overhead into consideration. In this paper, we propose a reinforcement learning (RL)-based beamforming scheme in a vehicle-to-infrastructure system, where we jointly determine the antenna beamwidth and the beam alignment interval, taking into account the past and future rewards. The simulation results show that the proposed RL-based joint beamforming scheme outperforms conventional beamforming schemes in terms of the average throughput and the average link stability ratio.

## 1. Introduction

As autonomous driving technologies evolve and application services become more advanced, autonomous vehicles generate more and more wireless data, which results in a huge strain on vehicle networks [1]. Hence, vehicle communication technologies are receiving great attention from both industry and academia [2,3]. The integration of 5G technology in vehicular communication systems aims to enhance the capabilities of connected vehicles and enable various advanced applications to improve road safety, traffic efficiency, and overall transportation systems [4,5]. One of the critical requirements for 5G vehicular communications is low latency. Researchers are working on developing communication protocols and technologies to minimize latency, ensuring quick and reliable data exchange between vehicles and infrastructure. In order to provide low latency and high data service to vehicles on the road, the integration of edge computing in 5G vehicular networks is gaining attention. Edge computing brings computational resources closer to the edge of the networks, such as base stations or road-side units (RSUs) [6,7]. Through multi-access edge computing (MEC), vehicles can simultaneously offload their tasks to the edge computing servers and obtain high-speed computing services. The authors of [6] proposed low-latency offloading scheduling for dependent tasks in MEC-enabled 5G vehicular networks. The authors of [7] developed a deep reinforcement learning (RL)-based non-orthogonal multiple access (NOMA)-assisted secure offloading for vehicular edge computing (VEC) networks in the presence of multiple malicious eavesdropper vehicles.

The deployment of 5G vehicular communications will deliver higher data rates, lower latency, higher reliability, and more devices to support a variety of intelligent transportation systems (ITS). Research on 5G-enabled ITS has been a major research area. In [8], 5G technologies for the quality of user experience in vehicular networks were explored. The authors of [9] provided a comprehensive overview of research on 5G vehicular applications, communication, and computing. The 5G vehicular communications leverage advanced antenna technologies like beamforming and massive multiple-input multiple-output (MIMO), which can improve the communication efficiency between vehicles and the infrastructure by focusing the signals directionally and increasing the spectral efficiency. The MIMO antenna design is important to apply MIMO technologies to vehicle communications. The authors of [10] presented a comprehensive review of MIMO antenna design approaches for 5G and beyond. The authors of [11,12] designed and developed antenna radiators for the sub-6 GHz 5G frequency band. Recently, the Internet of vehicles (IoV) have become a key enabler for future smart and connected transportation systems to create an intelligent and interconnected transportation ecosystem. Hence, it is necessary to develop an IoV antenna that can cover all the frequency bands for the long-term evolution (LTE) and the mid-band 5G systems. The authors of [13] designed a vehicular antenna by modifying the design of the Vivaldi antenna to support a broadband frequency range.

In [14], 5G leverages millimeter-wave (mmWave) frequencies to provide high data rate services with higher bandwidths and accommodates a greater number of simultaneous connections. However, mmWave vehicle communications are more susceptible to blockage by other vehicles and also suffer from higher path loss due to vehicles’ high mobility. Hence, highly directional antenna beamforming technologies are required to mitigate the path loss effects of mmWave links. The main lobe generated by directional beamforming generally has a narrower beamwidth, which concentrates the transmitted power in a specific direction, mitigating some of the propagation challenges associated with mmWave frequencies [15]. In addition, directional beamforming improves the signal-to-noise ratio (SNR) at the receiver by reducing interference from other beams [16].

In order to form a beam in the direction of a moving vehicle, beam alignment must be performed periodically. The beam alignment between the transmitting and receiving antennas requires sending and receiving pilot signals, which results in signal overhead. In particular, as the beamwidth becomes narrower, the directional gain of the antenna increases, but more frequent beam alignment is required because it is more prone to misalignment due to the vehicle’s mobility, and the beam alignment takes longer because many directions have to be searched [17]. Hence, it is important to find a compromise between the antenna beamwidth and the achievable data rate [17,18].

In this paper, we propose a joint consideration of the problems of determining the beamwidth and aperiodic beam alignment interval in order to maximize the throughput in highway vehicle-to-infrastructure (V2I) communication. The contributions of this paper are as follows. First, we propose aperiodic beam alignment for the directional beamforming in V2I communication. To the best of our knowledge, for the first time, we dynamically determine when to start the next beam alignment. That is, the proposed scheme dynamically adjusts the beam alignment interval according to the channel environment. Second, we formulate an optimization problem that aims to maximize the data rate by jointly controlling the antenna beamwidth and the timing of the next beam alignment. Third, we propose a Q-learning model to solve the optimization problem, where the reward is determined as a weighted sum of current and past data rates and expected future data rates.

The rest of this paper is organized as follows. Section 2 investigates the previous studies and analyzes the limitations. Section 3 describes the system model. Section 4 presents the proposed RL-based beamforming scheme, where the state, action, and reward of the RL are described in detail. Section 5 provides the simulation results, and Section 6 concludes this paper.

## 2. Related Work

Many researchers have endeavored to reduce the beam alignment overhead or optimize the antenna beamwidth in order to maximize the throughput in multiple antenna systems. Some researchers have focused on reducing the beam alignment overhead in mmWave communication systems [19,20,21,22,23,24]. The authors of [19] proposed a new training algorithm that allocates more training resources to the beams with higher beamforming gain. The proposed algorithm of [19] reduces the misalignment probability and thus reduces the beam alignment overhead. The authors of [20] proposed a novel beam alignment scheme that quickly finds a strong propagation path by exploring the angle-of-arrival and angle-of-departure domains. The authors of [21] proposed a low overhead analog beam selection scheme, where they steered two different-width beams: wide beams and narrow beams. They used a convolutional long short-term memory (LSTM) network to construct a narrow beam based on the wide-beam measurements. They reduced the time overhead by reducing the number of measurements. The authors of [22] developed an optimization problem of minimizing the average beam alignment overhead in mmWave networks with a large number of mobile users, where they formulated the problem as a constrained Markov decision process (CMDP). They showed the proposed heuristic algorithm based on the age information of users as approximate to the optimal solution. The authors of [23] proposed a sensor-aided beam-tracking strategy in vehicle-to-vehicle (V2V) communications. A sensor-aided control system, which allows the vehicles to exchange dynamic pose information, tracks highly direct beams without beam alignment procedures. Hence, the proposed sensor-aided beam-tracking does not require the beam-tracking overhead. The authors of [24] proposed a motion-sensor-based beam-tracking strategy. Based on the exchange of attitude information between the transmitter and the receiver, it steers the beam in the right direction without beam searching while the receiver moves.

Other researchers have focused on optimizing the beamwidth by taking into consideration the beam alignment overhead in order to maximize the throughput [17,25,26,27,28,29,30,31,32]. The authors of [17] addressed the beam alignment-throughput tradeoff and developed an optimization problem that jointly considered the problems of beamwidth selection and scheduling in a mmWave network with multiple transmitter–receiver pairs. The authors of [25] formulated an optimization problem to find the antenna beamwidth in V2I communication, taking into account the time consumption due to beam alignment. They used a recursive algorithm to solve the optimization problem. The authors of [26] formulated a distributed antenna beamwidth optimization problem in device-to-device (D2D) communication and used multi-agent deep RL in order to find the antenna beamwidth of each D2D pair. The author of [27] proposed a joint power and beamwidth optimization algorithm for a NOMA system with limited coherence time. They found the power and beamwidth that maximize the sum rate by iterating between the power allocation and the beamwidth optimization. The authors in [28] proposed a beamwidth-aware scheduling scheme for V2V communications, where the transmitter needs to transmit the same data to several neighboring vehicles at the same time by adapting the beamwidth confirmation. The authors of [29] formulated an optimization problem to jointly determine the beamwidth selection and power allocation in mmWave communication systems. They used the deep Q network (DQN) to solve the optimization problem. The authors of [30] proposed two beamwidth optimization methods to maximize the average throughput in V2V communications with inaccurate vehicular position information. The proposed beamwidth determination method improves the performance compared to the traditional beam-sweeping methods by considering localization errors in order to avoid beam misalignment. The authors of [31] investigated the beamwidth selection and power allocation problem in a NOMA mmWave system. They employed the NOMA scheme during the beam alignment phase and solved a convex optimization problem for maximizing the sum rate. The authors of [32] formulated mixed-integer nonlinear programming (MINLP) to maximize the network throughput and energy efficiency in mmWave heterogeneous networks, taking into account the tradeoff between alignment overhead and effective data transmission time. They proposed a novel outer approximation algorithm to solve the MINLP problem. However, in all the above previous work, beam alignment is periodically performed, which results in incurring periodic overhead.

## 3. System Model

### 3.1. System Description

**Figure 1:**
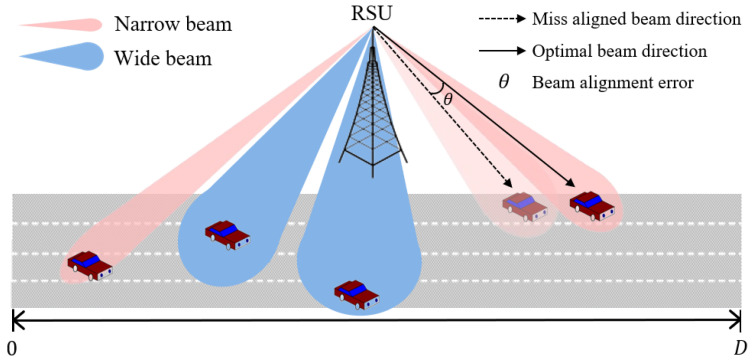
A system model.

We consider a highway downlink communication scenario with a single RSU and *K* vehicles, where the vehicles move along a multi-lane road at speed *v* in the positive direction of the x-axis, as shown in Figure 1. The communication range of the RSU is [0, D], where the communication range denotes the distance traveled by vehicles from when they start receiving service from the RSU on the road until they hand off to another RSU. We assume that there is no interference among *K* V2I links [33,34]. Since the vehicle moves, the beam alignment must be performed periodically or aperiodically between the RSU and the moving vehicles.

We assume that the RSU and vehicles are equipped with a directional antenna and are enabled to rotate the antenna bore-sight toward the desired direction. The RSU selects a beamwidth from the available set of beamwidths [35,36]. The directional antenna gain in the 3GPP Gaussian antenna model is given by [17,37]:

(1) $$\begin{array}{c} G\left(\theta \right)=\left\{\begin{array}{cc} G_{m}e^{-\rho \theta^{2}}, & |\theta |\leq \varphi \\ G_{s}, & \mathrm{otherwise}, \end{array}\right. \end{array}$$

where $\rho =2.028\ln(10)/\varphi^{2}$, 2φ is the main-lobe beamwidth, and θ denotes an alignment error relative to the antenna’s bore-sight direction. $G_{m}=\pi 10^{2.028}/\left(42.6443\varphi +\pi \right)$ and $G_{s}=G_{m}e^{-\rho \varphi^{2}}=10^{-2.028}G_{m}$ represent the maximum main-lobe gain and the side-lobe gain, respectively.

Several pilot transmissions and receptions are required to align the beam between the RSU and the vehicle. We assume a two-step hierarchical beam alignment method, where the first stage finds a sector-level beamwidth via an exhaustive search, and the second stage refines the beam search using narrower beam-level beamwidths in a subspace of the selectable sector-level beamwidths for all possible combinations [26,38]. For the *k*th V2I link, when $\psi^{k}$ denotes the sector-level beamwidth and $\varphi^{k}$ denotes the beam-level beamwidth, the number of possible combinations to be searched is ${\left\lceil \psi^{k}/\varphi^{k}\right\rceil }^{2}$. Without loss of generality, assuming the sector-level beamwidth alignment has been searched, the beam alignment time required to find the beam-level beamwidth can be expressed as follows [38]:

(2) $$\begin{array}{c} t_{a}^{k}\left(\varphi^{k}\right)={\left\lceil \frac{\psi^{k}}{\varphi^{k}}\right\rceil }^{2}\cdot T_{p}, \end{array}$$

where $T_{p}$ is the pilot transmission time. From (2), when the sector-level beamwidth is fixed, the narrower the beam-level beamwidth, the longer it takes exponentially to perform the beam alignment, which may result in the lower throughput due to the decrease in the data transmission time.

In a phased array antenna, the beamwidth can be adjusted via the control of the phase and the amplitude of the signals fed to each individual antenna element. By changing the relative phase of the signals fed to each antenna element, we can steer the beam in a particular direction. The steering changes the effective aperture of the array in the direction of interest, which in turn affects the beamwidth. Moreover, by applying specific amplitude weights to the individual elements, we can shape the radiation pattern and adjust the beamwidth. The number of antenna elements and the spacing between the elements also affect the beamwidth. Increasing the element spacing typically narrows the beamwidth, while decreasing the spacing widens it [39]. The authors of [40] proposed two types of beamwidth control methods based on conventional beamforming and Dolph–Chebyshev beamforming, for the cases with constant-modulus phase shifters and amplitude-adjustable phase shifters, respectively. The authors of [41] presented a beamforming technology utilizing transmission-line transformers and balanced impedance phase shifters. The impedance may vary while controlling the beamwidth due to changes in array configuration. However, the primary mechanisms used to control beamwidth, such as amplitude tapering, phase shifting, and steering, are generally designed to maintain a relatively constant input impedance across the operating frequency range. The authors of [41] investigated the S-parameters in a few common antenna configurations. The authors of [42] investigated the power loss according to the beamwidth and they observed that the power loss increases as the beamwidth narrows. Moreover, in order to narrow the beamwidth, the number of phase shifters needs to be increased, which may deteriorate the impedance matching due to the increased complexity and coupling in the phase shifters.

### 3.2. Frame Structure

**Figure 2:**
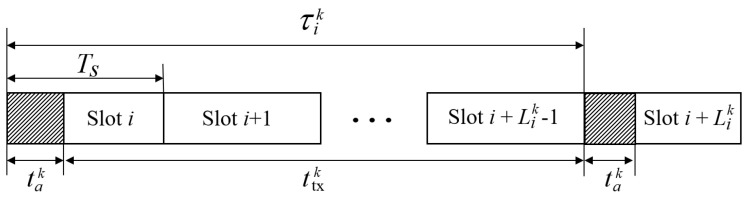
Time slot structure.

We consider a time-slotted frame structure with a slot duration of $T_{s}$, as shown in Figure 2. Consider the *k*th V2I link. After performing the beam alignment between the RSU and the vehicle at the beginning of the time slot *i*, the RSU transmits data to the vehicle during $t_{\mathrm{tx}}^{k}\left(\varphi_{i}^{k}\right)$ seconds, where $t_{\mathrm{tx}}^{k}\left(\varphi_{i}^{k}\right)$ is given by $\tau_{i}^{k}-t_{a}^{k}\left(\varphi_{i}^{k}\right)$, $\tau_{i}^{k}=L_{i}^{k}\cdot T_{s}$, and $t_{a}^{k}\left(\varphi_{i}^{k}\right)$, which is the beam alignment time, is obtained from (2). The beam alignment between the RSU and the vehicle proceeds again after $L_{i}^{k}$ time slots, i.e., $L_{i}^{k}$ is the beam alignment interval at time slot *i*. Hence, the parameters, namely the antenna beamwidth φ and the beam alignment interval *L*, affect on the throughput. Selecting a narrow antenna beamwidth results in higher antenna gain, but at the cost of a longer beam alignment time $t_{a}$ and a shorter beam alignment interval *L*. In other words, the narrower antenna beamwidth increases the received SNR at the vehicle but decreases the data transmission time.

The V2I communication link in a highway scenario is most likely to be a line-of-sight (LOS) link [43]. We use the WINNER+ channel model for modeling the path loss between the RSU and a vehicle. The WINNER+ channel model of [44] provides a comprehensive framework for modeling wireless communication channels in various scenarios. The WINNER+ channel models have been used by the 3GPP [45,46]. The path loss for the LOS channel in the WINNER+ channel model is as follows [44]:

(3) $$\begin{array}{c} \mathrm{PL}\left(d\right)=40.0\log_{10}\left(d\right)+7.56-17.3\log_{10}\left(h_{\mathrm{RSU}}\right)-17.3\log_{10}\left(h_{\mathrm{vehicle}}\right)+2.7\log_{10}\left(f_{c}\right), \end{array}$$

where $h_{\mathrm{RSU}}$ is antenna height of the RSU, $h_{\mathrm{vehicle}}$ is antenna height of the vehicle, *d* is distance, and $f_{c}$ is carrier frequency.

The received SNR can then be expressed as

(4) $$\begin{array}{c} {\mathrm{SNR}}^{k}=\frac{P_{\mathrm{tx}}G^{k}\left(\theta \right)\mathrm{PL}(d^{k})}{\sigma^{2}}, \end{array}$$

where $\mathrm{PL}(d^{k})$ represents the path loss at the distance $d^{k}$ between the RSU and the vehicle.

## 4. Proposed RL-Based Antenna Beamwidth and Beam Alignment Interval Optimization

### 4.1. Problem Formulation

Let $\lambda_{j}^{k}$ be the beam alignment indicator in the *k*th V2I link. If the beam alignment is performed at the beginning of time slot *j*, $\lambda_{j}^{k}=1$; otherwise, $\lambda_{j}^{k}=0$. Moreover, for the simplicity, we assume that the channel does not change during the slot time. The data rate at time slot *j* in the *k*th V2I link can then be expressed as follows:

(5) $$\begin{array}{c} R_{j}^{k}=\left(1-\lambda_{j}^{k}\frac{t_{a}^{k}\left(\varphi_{j}^{k}\right)}{T_{s}}\right)\cdot W\cdot \log_{2}\left(1+{\mathrm{SNR}}_{j}^{k}\right), \end{array}$$

where *W* is the system bandwidth, $\varphi_{j}^{k}$ is the antenna beamwidth updated in the beam alignment process, and ${\mathrm{SNR}}_{j}^{k}$, which is the received SNR at time slot *j*, is obtained from (4).

Our objective is to maximize the amount of data the vehicles receive from the RSU while moving along the road. The optimization problem that determines the beamwidth and beam alignment interval can then be expressed as follows:

(6) $$\begin{array}{cc} \max_{\Phi^{k},L^{k}}\; & \sum_{k=1}^{K}\;\sum_{j=1}^{N}\;S_{j}^{k}, \end{array}$$

where $S_{j}^{k}$, which is the amount of received data in time slot *j* in the *k*th V2I link, is given by $S_{j}^{k}=R_{j}^{k}T_{s}$; and $N^{k}$, which is the total number of time slots while the vehicle moves along the road, is given by $N^{k}=\left\lfloor D^{k}/\left(v^{k}T_{s}\right)\right\rfloor$. We need to find the optimal parameters, namely the set of beamwidths $\Phi^{k}=\left\{\varphi_{1}^{k},\cdots ,\varphi_{N^{k}}^{k}\right\}$ and the set of beam alignment intervals $L^{k}=\left\{L_{1}^{k},\cdots ,L_{N^{k}}^{k}\right\}$ for all *K* V2I links [47].

However, there exist tradeoffs among the antenna beamwidth, the beam alignment interval, and the amount of received data. The narrower the antenna beamwidth, the higher the received SNR of the vehicle, the shorter the beam alignment interval, and the shorter the data transmission time. Moreover, because the antenna beamwidth and beam alignment interval determined at the current time will affect the future performance, it is a noncausal system. For this reason, finding the optimal parameters is NP-hard; therefore, we propose an RL-based approach to solve the optimization problem.

### 4.2. Reinforcement Learning

**Figure 3:**
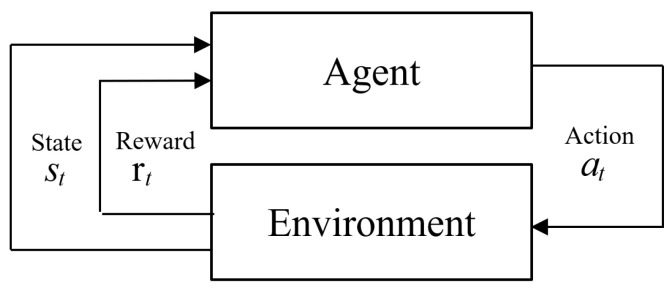
A general RL architecture.

A agent in RL observes the state in an environment that satisfies the Markov Decision Process (MDP) and takes an action according to the given policy. In return, the environment gives a reward to the agent and transit to a new state. Through these interactions, we obtain a sequence of states, actions, and rewards as $s_{0},a_{0},r_{1},s_{1},a_{1},r_{2},\cdots$. The goal of RL is aiming to learn the policy that yields maximal expected cumulative rewards within an episode, as follows [48]:

(7) $$\begin{array}{c} r_{t:T}=r_{t}+\gamma r_{t+1}+\gamma^{2}r_{t+2}+\ldots +\gamma^{T}r_{t+T-1}, \end{array}$$

where 0 ≤γ≤ 1 is the discount factor, *T* is the number of time steps, and $r_{t}$ is the reward obtained at time *t*. If γ=0, the agent is concerned with the immediate reward irrespective of the future rewards. The structure of the RL is shown in Figure 3.

To solve the decision problem of (6), we use a Q-learning model, which is a representative algorithm of the RL. The Q-learning model involves estimating the value of action for the state and managing these values as the Q-value in terms of the expected cumulative reward. The Q-value is stored in the Q-table as follows:

(8) $$\begin{array}{c} Q_{\pi }\left(s,a\right)=E\left[r_{t:T}|s_{t}=s,a_{t}=a,\pi \right]. \end{array}$$

The Q-table is iteratively updated via exploration and calculated using the Bellman equation as follows:

(9) $$\begin{array}{c} Q_{\pi }\left(s_{t},a_{t}\right)\leftarrow \left(1-\alpha \right)Q_{\pi }\left(s_{t},a_{t}\right)+\alpha \left[r_{t}+\gamma \max_{a}Q_{\pi }\left(s_{t+1},a\right)\right], \end{array}$$

where α∈(0,1] denotes the learning rate. The agent learns the policy $\pi^{*}$, when the Bellman optimality equation is satisfied as follows:

(10) $$\begin{array}{c} Q_{\pi^{*}}\left(s_{t},a_{t}\right)=\max_{a_{t+1}}\left(E\left[r_{t+1}+\gamma Q_{\pi^{*}}\left(s_{t+1},a_{t+1}\right)\right]\right). \end{array}$$

### 4.3. RL-Based Joint Antenna Beamwidth and Beam Alignment Interval Optimization

In the proposed RL-based joint optimization scheme, the state $s_{t}$ is defined as $s_{t}=\left(v_{t},d_{t}\right)$, where $v_{t}$ is the vehicle speed and $d_{t}$ is the distance traveled by the vehicle at time *t*. The action $a_{t}$ is defined as $a_{t}=\left(\varphi_{t},L_{t}\right)$, where $\varphi_{t}$ is the beamwidth and $L_{t}$ is the beam alignment interval at time *t*. The ϵ-greedy policy is used to select the action in order to balance exploration and exploitation. However, the action, the antenna beamwidth, and the beam alignment interval affect not only the amount of received data during the current time slot, but also the amount of received data while the vehicle moves along the rest of the road in the future. Consequently, the reward $r_{t}$ is defined as the weighted sum of the amount of data received in the past, $U^{\left(t-\right)}=\sum_{j=1}^{t-1}S_{j}$; the amount of data received during the current beam alignment interval, $U^{\left(t\right)}=\sum_{j=t}^{t+L_{t}-1}S_{j}$; and the amount of data received in the future, $U^{\left(t+\right)}=\sum_{j=t+L_{t}}^{N}S_{j}$.

(11) $$\begin{array}{c} r_{t}=\left(1-\beta \right)\cdot \left(U^{\left(t-\right)}+U^{\left(t\right)}\right)+\beta \cdot U^{\left(t+\right)}, \end{array}$$

where β is the weight for the future. However, because (11) is noncausal, we approximate the amount of data received in the future based on the received data in the current slot as $U^{\left(t+\right)}=\sum_{j=t+L_{t}}^{N}S_{t}$.

**Figure 4:**
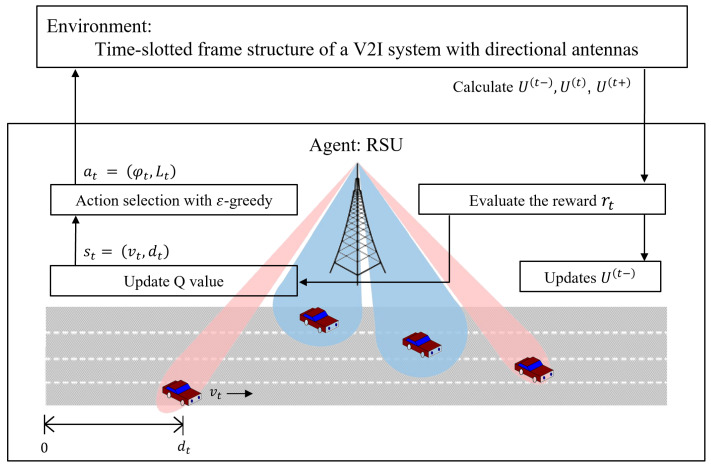
The structure of the proposed Q-learning-based joint optimization scheme.

Figure 4 shows the structure of the Q-learning-based joint antenna beamwidth and beam alignment interval optimization. The procedure of the algorithm is summarized in Algorithm 1. The Q-value and the amount of data received in the past are initialized with zero (line 1). The parameters, learning rate, discount rate, and weight for the future are configured (line 2). The system model is initialized as the environment and vehicles are generated on a multi-lane road (lines 4–5). The RSU agent obtains the state from the environment (line 7). The agent selects the beamwidth and the beam alignment interval as an action according to the ϵ-greedy policy (line 9). According to the action, the amount of received data during the current beam alignment interval is calculated (line 10). The number of remaining time slots and the amount of data to be received in the future is estimated based on the current beam alignment interval (lines 11–12). The reward is calculated and the amount of data received in the past is updated (lines 13–14). The Q-value and policy is updated via the above process, and the optimal policy is established at the end of each episode (lines 15–20).
**Algorithm** **1** Q-learning-based antenna beamwidth and beam alignment interval optimization  1: Initialize the Q-value function Q(*s*, *a*) and $U^{\left(t-\right)}$ with zeros;  2: Set the learning rate α, the discount rate γ, and the weight for the future β;  3: **for** each episode **do**  4:       Initialize environment;  5:       Generate *K* vehicles;  6:       **for** each vehicle **do**  7:             **for** each time slot **do**  8:                   The RSU get state $s_{t}$ from the environment;  9:                   The RSU selects an action $a_{t}$ based on the ϵ-greedy policy;10:                   Calculate $U^{\left(t\right)}$;11:                   Calculate the number of remaining time slot;12:                   Calculate $U^{\left(t+\right)}$;13:                   Calculate the reward $r_{t}$;14:                   Update $U^{\left(t-\right)}$;15:                   Update $Q_{\pi }\left(s_{t},a_{t}\right)$ by using (9);16:                   Update policy π17:             **end for**18:       **end for**19:       Update $Q_{\pi^{*}}\left(s_{t},a_{t}\right)$ by using (10);20:       Establish the optimal policy $\pi^{*}$;21: **end for**

The computation time of Algorithm 1 can be ignored because of the simplicity of the Q-learning model. In Algorithm 1, the RSU obtains the state, i.e., the speed and location of vehicles. After the RSU chooses an action, i.e., the beamwidth and beam alignment interval, it calculates the estimated data rate of the vehicle according to (5) and updates the Q-table. Hence, the computational time for each step can be ignored. However, in order to apply the proposed RL-based beamforming scheme, the training time is required to converge the Q-table.

## 5. Simulation Results

### 5.1. Simulation Environment

We evaluate the performance of the proposed scheme in a V2I downlink scenario with a single RSU and K=4 vehicles, where vehicles move at a constant speed *v*, on a 2 km road. The vehicles are randomly dropped on the different lanes between 0∼50 m on the road. The simulation parameters are summarized in Table 1. For simplicity of the RL model, we use the quantized antenna beamwidth and beam alignment interval time as follows: φ∈{5,6,⋯,15} [deg] and τ∈{1,2,⋯,5} [s].

For the performance comparison, we consider five schemes: the random selection scheme, the beamwidth optimization scheme, the beam alignment interval optimization scheme, and two joint beamwidth/interval optimization schemes. In the random selection scheme, both the beamwidth and the beam alignment interval are randomly selected for each time slot. In the beam alignment interval optimization scheme, the beam alignment interval is dynamically selected to maximize the amount of received data by using the RL for each time slot, but the antenna beamwidth is fixed to 7 degrees. Here, the value of the antenna beamwidth was chosen to maximize the performance of the beam alignment interval optimization scheme. In the beamwidth optimization scheme, the antenna beamwidth is dynamically selected to maximize the amount of received data by using the RL for each time slot, but the beam alignment interval time is fixed to 2 s [26]. Here, the beam alignment interval time was chosen to maximize the performance of the beamwidth optimization scheme. In joint beamwidth/interval optimization schemes, both the beamwidth and the beam alignment interval are dynamically selected for each time slot by using the RL. However, depending on whether the future reward in the RL model is taken into account, we classify the joint beamwidth/interval optimization schemes into the joint beamwidth/interval optimization scheme without future reward (i.e., β=0) and the proposed joint beamwidth/interval optimization scheme with future reward (i.e., β>0).

We first consider the performance metrics of the cumulative received data and the cumulative link stability time while the vehicle moves along the road. As long as the transmitter and receiver stay aligned in the main-lobe beamwidth, the link is stable or reliable, but while the transmitter and receiver stay aligned in the side-lobe beamwidth, the link may be in a low-quality state or may be disconnected. Hence, to satisfy the stable and high-quality service, we define the link stability time as the sojourn time in the antenna main lobe, i.e., $t_{\mathrm{tx}}^{k}\left(\varphi_{j}^{k}\right)-t_{\mathrm{sl}}^{k}\left(\varphi_{j}^{k}\right)$, at time slot *j* in the *k*th V2I link, where $t_{\mathrm{tx}}^{k}\left(\cdot \right)$ is the data transmission time and $t_{\mathrm{sl}}^{k}\left(\cdot \right)$ is the sojourn time in the antenna side lobe. The average link stability ratio of the *k*th V2I link can then be expressed as follows:

(12) $$\Gamma^{k}=\frac{1}{\sum_{j=1}^{N}\lambda_{j}^{k}}\sum_{j=1}^{N}\lambda_{j}^{k}\frac{t_{\mathrm{tx}}^{k}\left(\varphi_{j}^{k}\right)-t_{\mathrm{sl}}^{k}\left(\varphi_{j}^{k}\right)}{\tau_{j}^{k}},$$

where $\lambda_{j}^{k}$ is the beam alignment indicator and $\tau_{j}^{k}$ is the beam alignment interval time at time slot *j*, in the *k*th V2I link.

### 5.2. Impact of Weight for the Future Reward

**Figure 5:**
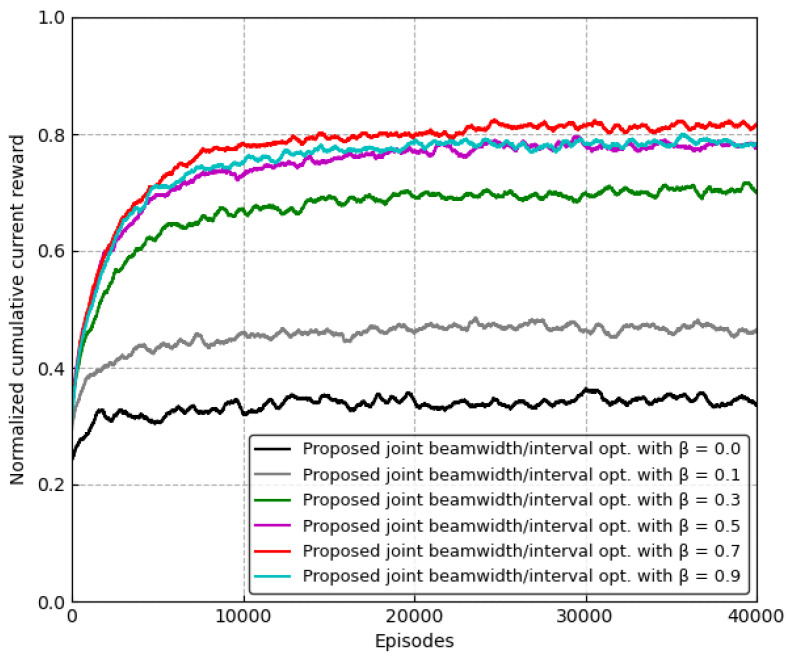
Convergence of the proposed scheme according to the different values of β.

Figure 5 shows the convergence of the proposed RL-based scheme for the different weights, β, in terms of the normalized cumulative reward $U^{\left(t\right)}$ over time (episodes). The proposed RL-based scheme converges as time passes. The current reward is the amount of received data during the current time and it depends on the β from (11). The performance of the proposed scheme is the worst when β=0, that is, it is important to take the future reward into account according to the beamwidth and the beam alignment interval. As the value of β increases, the performance increases, but when β becomes larger than 0.7, the performance decreases. From the simulation result, we set the weight for the future as β=0.7.

### 5.3. Performance of RL-Based Joint Beamforming Scheme

**Figure 6:**
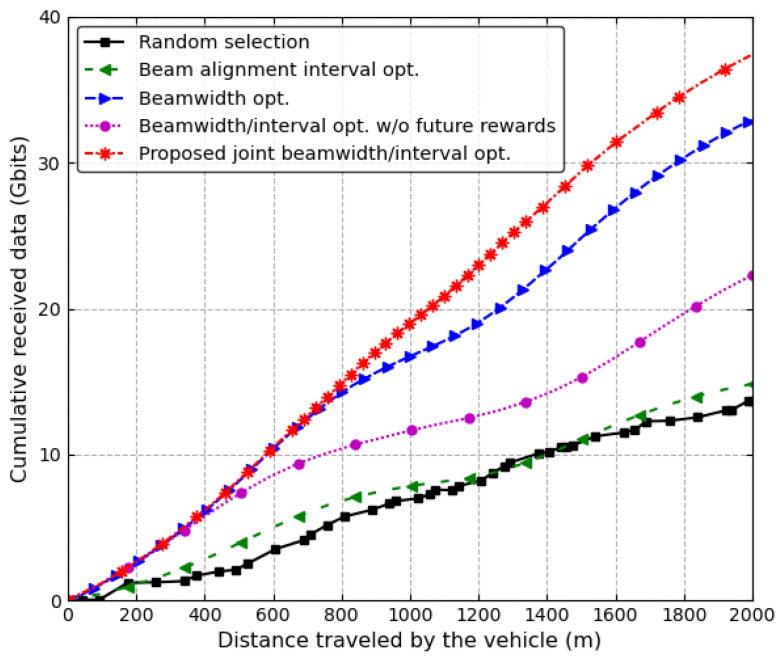
The cumulative received data of the vehicle on the first lane when v=60 km/h.

Figure 6 shows the cumulative received data of a vehicle on the first lane while four vehicles are moving along the different lanes at a speed of 60 km/h. The transmit power of the RSU is 24 dBm. In the proposed joint beamwidth/interval optimization scheme, the received data linearly increases; however, in the other schemes, the received data does not linearly increase. In particular, as the vehicle passes near the midpoint of the road, the performance deteriorates further. The random selection and beam alignment interval optimization schemes showed almost similar performance, being the worst performance. That is, if the beamwidth is randomly selected or fixed, the performance significantly deteriorates. Hence, it is essential to optimize the beamwidth as the vehicle moves. The proposed joint beamwidth/interval optimization scheme outperforms the other schemes because it adjusts both the antenna beamwidth and the beam alignment interval for each time slot, taking future rewards into consideration. However, if the future rewards are not considered, the performance of the proposed scheme would become inferior to the conventional beamwidth optimization scheme.

**Figure 7:**
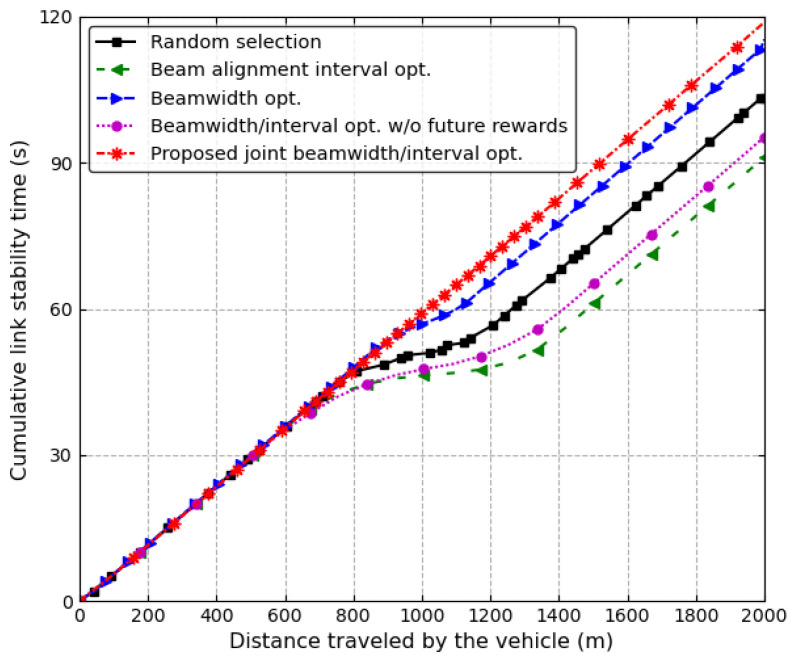
The cumulative link stability time of the vehicle on the first lane when v=60 km/h.

Figure 7 shows the cumulative link stability time of a vehicle on the first lane while four vehicles are moving along the different lanes at a speed of 60 km/h. The transmit power of the RSU is 24 dBm. In the proposed joint beamwidth/interval optimization scheme, the link stability time linearly increases, but in the other schemes, the link stability time significantly decreases at the midpoint of the road. In the middle of the road close to the RSU, the beamwidth needs to be widened to keep the same beam alignment time at the road edge. Although the antenna gain of a wide beamwidth is smaller than that of a narrow beamwidth, because the path loss is small at points close to the RSU, the data rate does not decrease significantly. Additionally, because the beam alignment time is short for a wide beamwidth, the beam alignment can be performed frequently to keep the main-lobe beamwidth. Hence, when a vehicle passes near the midpoint of the road, the proposed scheme can increase the link stability time and throughput by adjusting both the antenna beamwidth and the beam alignment interval, but other schemes deteriorate the performance because they cannot control the antenna beamwidth and the beam alignment interval simultaneously. In particular, the beam alignment interval optimization scheme with a fixed beamwidth shows the worst performance because the beamwidth cannot be adjusted near the midpoint of the road.

**Figure 8:**
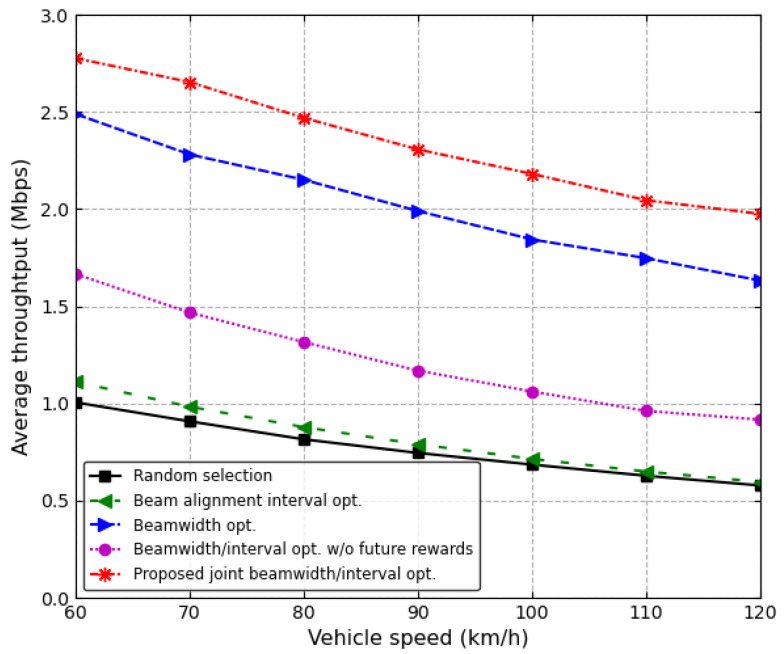
Average throughput vs. vehicle speed.

**Figure 9:**
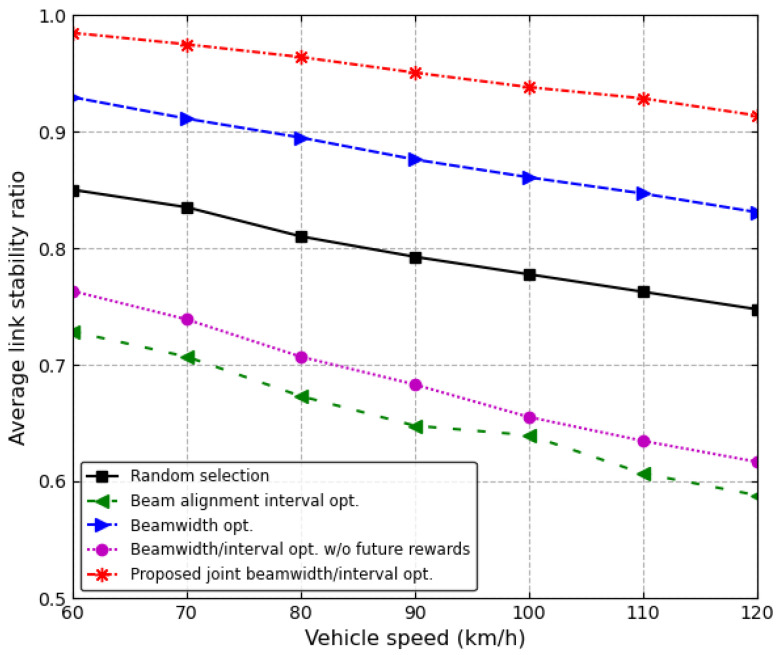
Average link stability ratio vs. vehicle speed.

Figure 8 and Figure 9 show the average throughput and the average link stability ratio over the entire time that four vehicles are moving on the road. The transmit power of the RSU is 24 dBm. Figure 8 shows the average throughput as the speed of vehicles increases. As the vehicle speed increases, the beam should be frequently aligned due to beam alignment errors, and therefore, the throughput decreases with the vehicle speed. Moreover, as the vehicle speed increases, the time in which the vehicle leaves the road becomes shorter; therefore, the amount of received data from the RSU decreases. Consequently, as the vehicle speed increases, the average throughput decreases for all of the schemes. The proposed joint beamwidth/interval optimization scheme outperforms other schemes. In particular, when the vehicle speed is 90 km/h, the proposed scheme increases the average throughput by about 15.9% and 98.8% in comparison with the conventional beamwidth optimization scheme and joint beamwidth/interval optimization scheme without future reward, respectively.

Figure 8 shows the average link stability ratio of a vehicle on the first lane as the speed of four vehicles increases. As the vehicle speed increases, beam alignment errors frequently occur, which results in a decrease in the link stability time, i.e., the sojourn time in the antenna main lobe. Since the proposed scheme dynamically controls the beam alignment interval as well as the antenna beamwidth, the proposed scheme can increase the link stability time. Hence, the proposed joint beamwidth/interval optimization scheme outperforms other schemes in terms of the average link stability ratio. The beam alignment interval optimization scheme with a fixed beamwidth shows the worst average link stability ratio because the fixed narrow beamwidth increases the beam alignment errors with the vehicle speed. In particular, when the vehicle speed is 90 km/h, the proposed scheme increases the average link stability by about 8.6% and 24.8% in comparison with the conventional beamwidth optimization scheme and the random selection, respectively.

**Figure 10:**
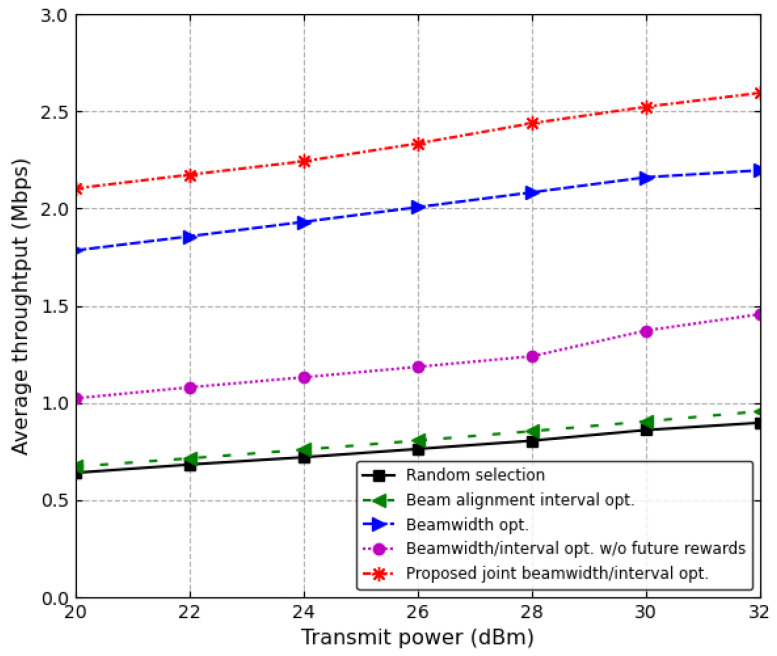
Average throughput vs. transmit power.

**Figure 11:**
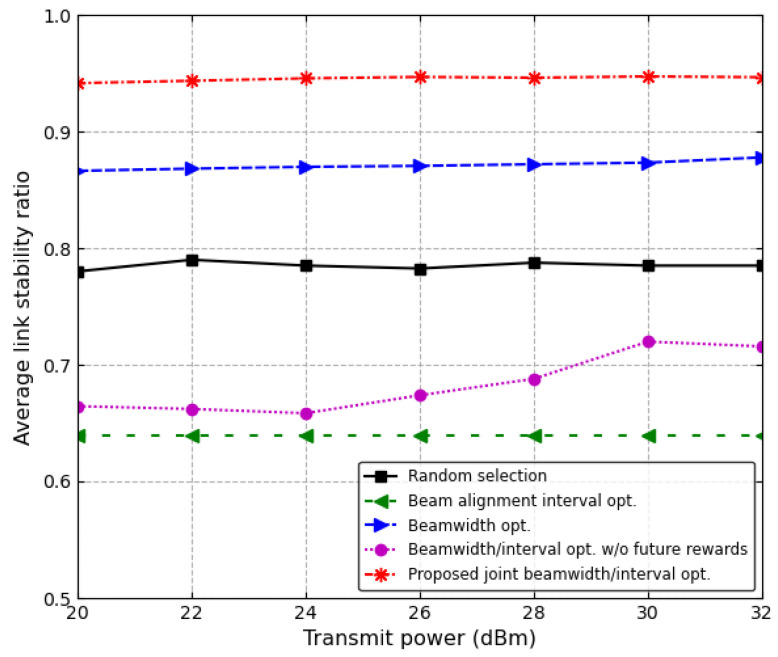
Average link stability ratio vs. transmit power.

Figure 10 and Figure 11 show the average throughput and the average link stability ratio according to the transmit power of the RSU, respectively, where the speeds of four vehicles, in order of the vehicles closest to the RSU, are 80 km/h, 90 km/h, 100 km/h, and 110 km/h, respectively. Figure 10 shows the average throughput as the transmit power of the RSU increases. The received SNR at each vehicle increases according to the transmit power of the RSU. Hence, as the transmit power of the RSU increases in dB scale, the average throughput almost linearly increases for all of the schemes. In particular, when the transmit power of the RSU is 20 dBm, the proposed scheme increases the average throughput by about 17.9% and 105.5% in comparison with the conventional beamwidth optimization scheme and joint beamwidth/interval optimization scheme without future reward, respectively.

Figure 11 shows the average link stability ratio as the transmit power of the RSU increases. The average link stability ratio depends on both the beam alignment interval and the beamwidth. As the beam alignment interval becomes longer or the beamwidth becomes narrower, the vehicle will deviate from the main lobe of the beam faster and stay longer in the side lobe of the beam. That is, the average link stability ratio depends on the method of determining the beam alignment interval and beamwidth. In the random selection scheme, both the beam alignment interval and the beamwidth are randomly selected; therefore, the average link stability ratio remains constant regardless of the transmit power. Moreover, for all schemes expect the joint beamwidth/interval optimization scheme without future reward, because the beam alignment interval or the beamwidth is optimally selected, the average link stability ratio exhibits approximately a constant value regardless of the transmit power. However, in the joint beamwidth/interval optimization scheme without future reward, the beam alignment interval and beamwidth are not optimized. Since the scheme tries to increase the instantaneous received power at the vehicle, if the transmit power of the RSU is low, a narrow beamwidth is selected; therefore, the sojourn time in the main lobe decreases. On the other hand, as the transmit power of the RSU increases, a wider beamwidth is selected, which increases the sojourn time in the main lobe. However, if the transmit power is too small or too large, the sojourn time in the main lobe becomes independent of the transmit power. Hence, in the joint beamwidth/interval optimization scheme without future reward, the average link stability ratio linearly increases as the transmit power of the RSU increases within a certain range, [24 dBm, 30 dBm]. The proposed joint beamwidth/interval optimization scheme outperforms other schemes. In particular, when the transmit power of the RSU is 20 dBm, the proposed scheme increases the average link stability by about 8.7% and 21.9% in comparison with the conventional beamwidth optimization scheme and the random selection scheme, respectively.

## 6. Conclusions

In vehicle communications, the directional antenna combined with beamforming is the key technology to satisfy the quality-of-service of advanced autonomous vehicles. To maximize the throughput due to beamforming, it is important to manage the signaling overhead required to control the beamwidth and beam alignment. In this paper, we proposed a joint antenna beamwidth and beam alignment interval optimization scheme in highway V2I communication. We designed an aperiodic beam alignment strategy to overcome the signaling overhead caused by the periodic alignment procedure and formulated the optimization problem that jointly determines the beamwidth and the beam alignment interval. Moreover, to overcome the noncausal problem, we approximated the future reward and used a Q-learning model to solve the optimization problem. The developed Q-learning model jointly determines two parameters of the proposed scheme, namely antenna beamwidth and beam alignment interval, where the reward is defined as the weighted sum of the past and expected future rewards. The proposed RL-based joint beamwidth/interval optimization scheme outperforms the other schemes. When the vehicle speed was 90 km/h, the proposed scheme increased the average throughput by about 15.9% and 98.8%, and increased the average link stability by 8.6% and 24.8% in comparison with the conventional beamwidth optimization scheme and the joint beamwidth/interval optimization scheme without future reward, respectively. The proposed aperiodic beam alignment scheme for directional beamforming increases the average throughput and link stability; moreover, it can decrease the beam alignment overhead.

In this paper, for simplicity of the environment, we assumed that the vehicles are located at random distances in different lanes of the road and move at a constant speed. Additionally, there was assumed to be no interference among V2I links. Hence, if the initial positions of the vehicles are sufficiently far away, the beamwidth and beam alignment interval of the V2I link can be determined without considering the positions of other vehicles. However, in a practical environment, when the RSU steers the beam in a particular direction and determines the beamwidth, it should consider the positions of other vehicles to avoid the inter-beam interference [49,50]. For example, if a serving vehicle is close to subsequent vehicles or vehicles in other lanes, the RSU would select a narrow beamwidth to avoid the inter-beam interference, even though the beam alignment overhead is increased. Unfortunately, the optimization problem of determining the beamwidth and beam alignment interval of all vehicles on the road, taking into account their locations and inter-beam interference as well as the channel state of all vehicles, is an NP-hard problem. Second, the variable speed of the vehicle makes uncertainties in the practical environment. When a vehicle varies its speed while moving on the road, it is difficult to predict the amount of data it will receive in the future until the vehicle hands off to other RSU, and it is also difficult to predict the inter-beam interference with other vehicles. For example, if a subsequent vehicle suddenly accelerates, it leads to the increased beam interference with the preceding vehicle. Moreover, the uncertainty increases the complexity and convergence time of the machine learning model.

This paper assumed no inter-beam interference and that vehicles move at a constant speed, but the trends in performance results are expected to be similar even in the practical environment. For further study, this work can be extended to a V2I network with inter-beam interference. If the received SNR is modified to take the inter-beam interference into consideration and deep reinforcement learning is applied, the proposed approach can be extended to find a global solution in a practical environment.

## Figures and Tables

**Table 1:** Simulation parameters.

Parameter	Value
Carrier frequency	5.9 GHz
Channel bandwidth	1 MHz
Noise power spectral density	−174 dBm/Hz
Slot duration	0.01 s
Pilot transmission time	0.001 s
Path loss model	WINNER+B1, LOS
V2I shadowing model	Log-normal with $\sigma^{2}=3$ dB
Antenna height of the RSU	10 m
Antenna height of each vehicle	1.5 m
Learning rate	0.99
Discount factor	0.01
Greedy rate	0.1

## Data Availability

Data are contained within the article.
